# Neutrophil membrane biomimetic delivery system (Ptdser‐NM‐Lipo/Fer‐1) designed for targeting atherosclerosis therapy

**DOI:** 10.1049/nbt2.12137

**Published:** 2023-05-15

**Authors:** Wei Li, Chang Liu, Sichuan Wang, Naifeng Liu

**Affiliations:** ^1^ Department of Cardiology Zhongda Hospital School of Medicine Southeast University Nanjing China; ^2^ Department of Cardiology Affiliated Hospital of Yangzhou University Yangzhou University Yangzhou China; ^3^ Department of Pharmaceutics State Key Laboratory of Nature Medicines China Pharmaceutical University Nanjing China

**Keywords:** atherosclerosis, biomimetic delivery system, drug targeting, ferroptosis, neutrophil

## Abstract

Atherosclerosis is a progressive inflammatory disease characterised by excessive lipid accumulation and inflammatory cell infiltration and is the basis of most cardiovascular diseases and peripheral arterial diseases. Therefore, an effectively targeted delivery system is urgently needed to deliver ferroptosis‐specific inhibitors to the site of arterial plaque and the inflammatory microenvironment. Inspired by the fact that neutrophils can be recruited to arterial plaques under the action of adhesion molecules and chemokines, the authors developed a neutrophil membrane hybrid liposome nano‐mimetic system (Ptdser‐NM‐Lipo/Fer‐1) that delivers Ferrostatin‐1 (Fer‐1) to the atherosclerotic plaque effectively, which is composed of Fer‐1‐loaded Ptdser‐modified liposomes core and neutrophils shell. Fer‐1 was released at the AS plaque site to remove reactive oxygen species (ROS) and improve the inflammatory microenvironment. In vitro ROS clearance experiments have shown that 50 μmol/ml Fer‐1 can significantly remove ROS produced by H_2_O_2_‐induced MOVAS cells and Ptdser‐NM‐Lipo/Fer‐1 revealed a 3‐fold increase in the inhibition rate of ROS than free Fer‐1 in induced‐RAW264.7, demonstrating its superior ROS‐cleaning effect. Based on the interaction of adhesion molecules, such as vascular cell adhesion molecule 1, ICAM‐1, P‐selectin, E‐selectin, and chemokines released in the inflamed site, the aorta in NM‐Lipo‐treated mice displayed 1.3‐fold greater radiant efficiency than platelet membrane‐Lipo‐treated mice. Meanwhile, due to the modification of the Ptdser, the aorta in Ptdser‐NM‐Lipo/Fer‐1‐treated mice exhibited the highest fluorescence intensity, demonstrating its excellent targeting ability for atherosclerosis. Therefore, we present a specific formulation for the treatment of atherosclerosis with the potential for novel therapeutic uses.

## Graphical abstract

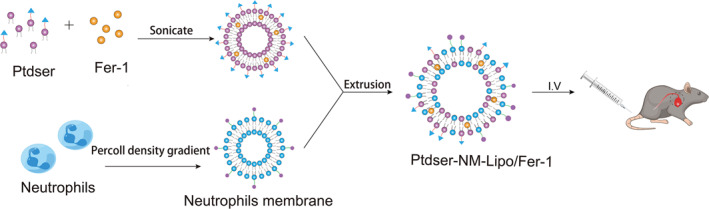

**Abbreviations**
AS: atherosclerosis
DCFH‐DA: 2′,7′‐dichlorodihydrofluorescein
DMSO: methyl sulfoxide
eNOS: nitric oxide synthase
Fer‐1: ferrostatin‐1
ICAM‐1: intercellular adhesion molecule 1
LDL: low‐density lipoprotein
Lipo: liposome
LPS: lipopolysaccharide
MTT: 3‐ (4,5)‐dimethylthiahiazo (‐z‐y1)‐3,5‐di‐ phenytetrazoliumromide
NM: neutrophil membrane
Nrf2: nuclear factor E2‐related factor 2
ox‐LDL: oxidised low‐density lipoprotein
PEG: polyethylene glycol
PM: platelet membrane
PtdSer: phosphatidyl phosphatidylserine
ROS: reactive oxygen species
TLR: Toll‐like receptor
VCAM‐1: vascular cell adhesion molecule 1

## 1 INTRODUCTION

Atherosclerosis (AS)‐related cardiovascular disease is the leading cause of increased morbidity and mortality worldwide. The accumulation of low‐density lipoprotein (LDL) cholesterol under the arterial intima caused by cardiovascular risk factors such as hypertension and diabetes is the main culprit of AS [1]. When atherosclerotic plaques begin to form, circulating apolipoprotein B containing lipoproteins enter the endothelial space, where they were modified and recognized by innate immune cells as danger signals. These danger signals activate Toll‐like receptor signalling and inflammasomes in innate immune cells, triggering an inflammatory response that includes cytokine production and secretion, reactive oxygen species (ROS) generation, up‐regulation of costimulatory molecules and P‐selectin (P‐Selectin), E‐selectin (E‐Selectin), intercellular adhesion molecule 1 (ICAM‐1), and vascular cell adhesion molecule 1 (VCAM‐1), which recruit monocytes to atherosclerotic plaques. Macrophages from monocyte differentiation could take up lipoproteins in plaques such as oxidised low‐density lipoprotein, followed by transforming to lipid‐rich foam cells and forming the necrotic core of plaques [2, 3, 4].

Therefore, macrophages are the main immune cells in AS, and the oxidation of LDL, phagocytosis, and foam cell formation by macrophages all play a crucial role in the formation of AS plaques [5]. Foamy macrophages undergo ferroptosis and release cellular components and lipids to form a necrotic core, which is one of the key pathological progressions in AS. Ferroptosis is a novel type of programmed cell death mediated by iron‐dependent lethal lipid peroxidation and is morphologically characterised by loss of plasma membrane integrity, swelling of the cytoplasm and organelles, malformed atrophic mitochondria with loss of cristae, condensation, and the adventitia ruptured [6]. Atherosclerotic plaques have a pro‐inflammatory M1 macrophage phenotype and an anti‐inflammatory M2 macrophage phenotype. Among them, the low expression of ferroportin in M1 macrophages promotes the occurrence and development of AS. The increase in free iron promotes inflammation and the formation of macrophage‐derived foam cells followed by promoting ROS accumulation, lipid peroxidation, and plaque haemorrhage, indicating that macrophage ferroptosis is involved in the occurrence and development of AS [7].

Ferrostatin‐1 (Fer‐1) was a pharmacological compound identified as an effective ferroptosis‐specific inhibitor, which could prevent the accumulation of lipid ROS, inhibit lipid peroxidation, and reduces labile iron in cells. Recent studies have shown that Fer‐1 could down‐regulated the expression of prostaglandin‐endoperoxide synthase 2 and up‐regulated the expression of GPX4 and nuclear factor E2‐related factor 2 (Nrf2) proteins, and protect cells by inhibiting oxidative stress and reducing ROS generation [8]. However, as a promising pharmacological molecule, Fer‐1 faced the issues of inherent stability and hydrophobicity, which limited its application in vivo.

Treatments for atherosclerotic plaque regression have been broadly divided into dietary and lifestyle interventions and drug therapy. However, due to the non‐specific distribution of drugs, the accumulation of drugs in atherosclerotic plaques is low, and unexpected biodistribution leads to damage to healthy tissues. Nanotechnology has been widely applied in drug delivery systems design and has been considered as a hotspot in the research field of international pharmacy [9, 10, 11].

Recently, biomimetic drug delivery systems with a natural cell membrane as the shell and synthetic drug‐loaded nanoparticles as the core has attracted great attention from researchers [12]. The structure and functionality of the cell membrane, in particular the unique functional proteins on the cell membrane surface, are maintained by this tactic. Even though nanotechnology brings a revolution in drug delivery systems’ development, there are still some issues that need to be solved for AS treatment: 1) The efficiency of targeted delivery using only biomimetic cell membrane‐modified carriers is not high, and the dose of drugs distributed to the AS site is small, resulting in poor therapeutic effect; 2) Using antibodies or antibody fragments as active targeting ligands to modify the carrier, there are problems such as large molecular weight, poor stability, and potential immunogenicity; 3) Lipoprotein receptor targeted nanocarriers targeting AS plaques also leading to the accumulation of in the liver due to the wild expression of their receptors [13]. Therefore, how to use the biomimetic carrier system to safely and effectively target atherosclerotic plaque and carry out precise drug delivery is the key and difficult point that needs to be solved urgently.

Phosphatidyl phosphatidylserine (PtdSer) is an important biofilm widely present in bacterial, yeast, plant, and mammalian cells. In apoptotic cells, PtdSer flips outward to the exterior of the cell membrane and is exposed to the extracellular environment, resulting in increased exposure to the surface of apoptotic bodies. Increased surface exposure of apoptotic bodies releases key ‘eat‐me’ signals, thereby enabling effective recognition and phagocytosis by macrophages in vivo [14], which induce macrophages to secrete anti‐inflammatory mediators such as TGF‐β and IL‐10 but decrease the secretion of the pro‐inflammatory factor such as TNF‐α and IL‐1β. Studies have shown that nanocarriers modified with phagocytosis signal PtdSer can target inflammatory macrophages in atherosclerosis and obesity in vivo. Therefore, PtdSer‐modified nanocarriers can actively target inflammatory macrophages in AS lesions, and be taken up by macrophages to exert anti‐inflammatory effects, which exhibit potential in the active targeting therapy of AS.

Currently, there are two main categories of medication delivery systems that can be used to treat AS both domestically and internationally: passive targeting and active targeting. Due to the AS plaque site's expanded endothelium intercellular space, enhanced vascular permeability, and incomplete structure, nanoparticles of the right size can passively target the AS site. Second, several distinct sick cells or tissues that are present during AS can be employed as targets for active nano delivery system targeting. For example, platelet membranes [15] were used in the diagnosis of atherosclerosis along with the adhesion of platelets and collagen exposed to the surface of the damaged endothelium, but neutrophil membranes have a stronger chemotaxis effect at the site of inflammation which was one of the most prevalent leucocyte and one of the first to get to inflamed tissue. When neutrophils are activated, they can form neutrophil extracellular traps, a well‐defined release mechanism that allows neutrophils to act as drug or nanocarrier‐loaded delivery systems early in inflammation. IGG‐loaded neutrophil membrane biomimetic NPs [16] have been shown in studies to target inflammatory and high‐risk atherosclerotic plaques in vivo and assess therapeutic efficacy.

Thus, the combination of Ptdser‐modified liposome and NM can not only innovatively target the inflammatory based on the development of atherosclerosis, but also can rely on its immune properties to effectively avoid phagocytosis of the hepatosplenic reticular endothelial system, to realise the function of long‐term blood circulation, and ultimately significantly improve the therapeutic effect.

Herein, we constructed a novel NM‐based biomimetic delivery system (Ptdser‐NM‐Lipo/Fer‐1) for precisely targeted therapy of AS. After intravenous injection of Ptdser‐NM‐Lipo/Fer‐1, the adhesion molecules such as VCAM‐1, ICAM‐1, P‐selectin, E‐selectin, and chemokines released during AS inflammation could help PtdSer‐NM‐Lipo/Fer‐1 targeted and enriched in the lesions of AS. Recognition and phagocytosis by macrophages due to the Ptdser modification improved the targeting efficiency of PtdSer‐NM‐Lipo/Fer‐1 to AS lesions. The released Fer‐1 from the nanocarriers strongly inhibits the accumulation of unstable iron and ROS and upregulates the expression of nitric oxide synthase by regulating the expression of SLC7A11 and GPX4, thereby inhibiting the development of AS and ferroptosis. The results of in vitro and in vivo experiments proved that this system could effectively inhibit the formation and development of AS. Ptdser‐NM‐Lipo/Fer‐1 could be used as an effective treatment for AS and has the prospect of clinical research.

## 2 MATERIALS AND METHODS

### 2.1 Materials and cell culture

Phosphatidylcholines, cholesterol, and Ferrostatin‐1 (>98%) were purchased from Aladdin Reagent Co., Ltd. (Shanghai, China). Dioleoyl phosphatidylserine (PtdSer) was purchased from A.V.T. Pharmaceutical Co., Ltd. (Shanghai, China). All other chemicals and reagents were analytical grade. ApoE^−/−^ mice (6 weeks old, 18–22 g) were purchased from Qinglongshan Animal Breading Centre (Nanjing, China).

MOVAS cells and RAW 264.7 cells were cultured in Dulbecco's Modified Eagle Medium (DMEM medium) with 5% foetal bovine serum (FBS) in a T25 flask and incubated at 37 °C in a humidified atmosphere containing 5% CO_2_. As the culture reaches 80%–90% confluency, the medium was aspirated and the cells were rinsed with PBS followed by the addition of 1 mL trypsin‐EDTA and 5 min incubation at 37 °C to release cells. When 90% of the cells have detached, the equivalent of 3 volumes of pre‐warmed complete growth medium was added to neutralise the trypsin enzyme, and all of the liquid plus cells were transferred to a 15 ml tube for 3 min centrifugation at 1000 rpm. The cell pellet was resuspended and transferred to a new T25 flask.

### 2.2 Neutrophil membrane extraction

To isolate neutrophils through Percoll density gradient centrifugation, the whole blood of the mouse was carefully placed over a two‐layer per‐coll gradient of 65% and 55% and centrifuged at 400 g for 30 min in a 10 ml tube. The neutrophils were collected from the interface between 55% and 65% Percoll and suspended in 0.25 × PBS for 20 min at 4 °C to remove cellular contents, such as DNA or organelles. Neutrophil ghost cells were then pelleted by centrifugation at 6000 rpm for 10 min, following which the supernatant was discarded and the ghost was washed with 0.25 × PBS until the supernatant turned transparent. The protein concentration was quantified using a bicinchoninic acid assay.

### 2.3 Synthesis of Ptdser‐NM‐Lipo/Fer‐1

**Scheme 1:**
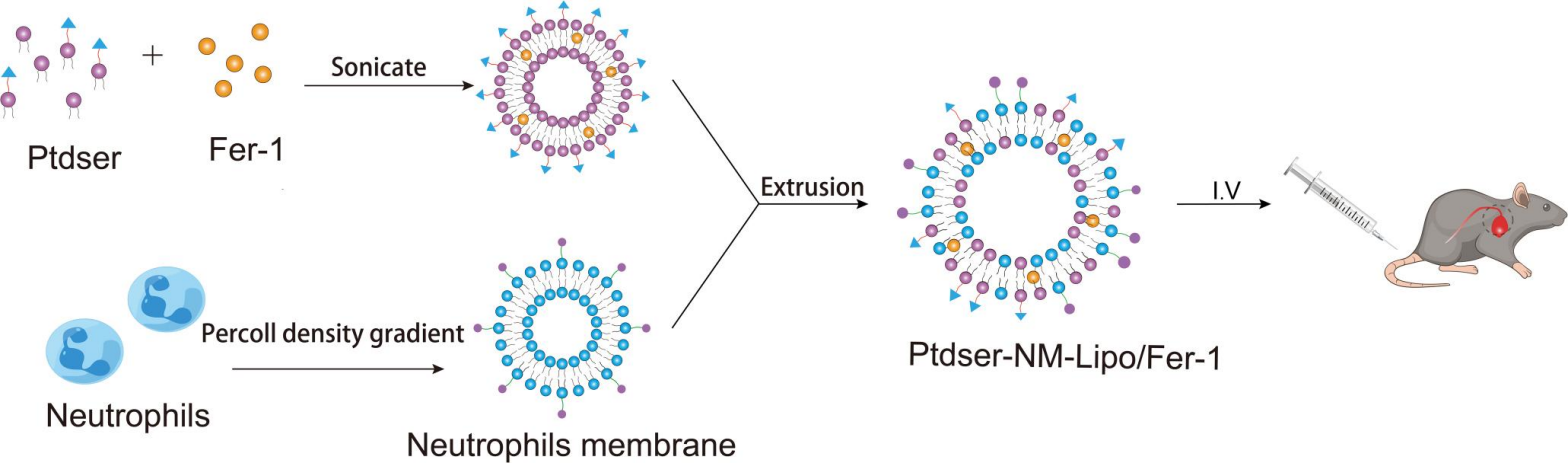
The synthesis of Ptdser‐NM‐Lipo/Fer‐1.

As shown in Scheme 1, 100 mg of phosphatidylcholines, 20 mg of cholesterol, and 100 μL Fer‐1 solution were added to 30 ml chloroform and dissolved completely in an eggplant bottle. The mixture was evaporated using a rotary evaporator under reduced pressure until a homogeneous phospholipid film formed which was hydrated with 3 ml PBS. The mixture was ultrasonicated and filtered by 0.22 μm‐filter to obtain homogeneous Fer‐1 loaded liposome (Fer‐1‐Lipo). After that, the liposome was mixed with PtdSer diluted in saline, then homogenised in an ice bath using ultrasonic probes at 30% power for 5 min to obtain Ptdser‐ Lipo/Fer‐1. For membrane coating, the NM was mixed with the liposome at a weight ratio of 30:1 (polymer‐to‐membrane protein) and the mixture was extruded serially through 400 nm and then 200 nm polycarbonate porous membranes to obtain the NM coated liposome (Ptdser‐NM‐Lipo/Fer‐1).

### 2.4 Characterisation of Ptdser‐NM‐Lipo/Fer‐1

The hydrodynamic diameters and zeta potentials of Ptdser ‐NM‐Lipo/Fer‐1 were measured using Malvern Laser Particle Size Analyse. The morphology of the Ptdser‐NM‐Lipo/Fer‐1 was evaluated by transmission electron microscopy (TEM). In brief, the diluted Ptdser‐NM‐Lipo/Fer‐1 was placed on a copper grid coated with carbon film and negatively stained by 1% phosphotungstic acid solution for 5 min, following that the vesicle was dried by infrared lamps and subjected to TEM observation.

### 2.5 Reactive oxygen species scavenging experiment

To evaluate the ROS‐cleaning effect of Fer‐1 for oxidised stress in smooth muscle cells and macrophages, MOVAS cells and RAW 264.7 cells were chosen as the cell models.

MOVAS cells were stimulated with different concentrations of hydrogen peroxide (250, 500, and 1000 μmol/L), and RAW264.7 cells were induced with lipopolysaccharide (LPS). In brief, MOVAS cells were seeded at densities of 1 × 10^5^ cells/mL into a 24‐well cell culture plate and divided into three groups with different treatments: (I) control group (DMEM medium); (II) Fer‐1@ H_2_O_2_ group (with the Fer‐1 concentration of 10,50 and 100 μmol/L); (III) Fer‐1& H_2_O_2_ group (with the Fer‐1 concentration of 10,50 and 100 μmol/L). For the Fer‐1@ H_2_O_2_ group, the cells were stimulated with H_2_O_2_ for 24 h followed by the addition of Fer‐1 solution for another 24 h. For the Fer‐1 & H_2_O_2_ group, cells were incubated with H_2_O_2_ and Fer‐1 solution for 24 h together. After a 24 h or 48 h incubation period, the content of ROS was measured.

Meanwhile, to compare the ROS‐cleaning effects of Ptdser‐NM‐Lipo/Fer‐1 and free fer‐1 solution in macrophages, Raw 264.7 cells were seeded in 24‐well plates at a density of 1 × 10^5^ per well and treated with LPS for 12 h. The LPS‐induced cells were divided into three groups: (I) control group; (II) Fer‐1 group; (II) Ptdser‐NM‐Lipo/Fer‐1 group (with the Fer‐1 concentration of 2, 5 and 10 μmol/L). For groups (II) and (III), the LPS‐induced cells were incubated with Fer‐1 or Ptdser‐NM‐Lipo/Fer‐1 for 24 h, respectively, after which the ROS content and ROS inhibition rate were measured.

The ROS measurement was measured using the probe 2′,7′‐Dichlorodihydrofluorescein (DCFH‐DA). After incubation, the medium was pipetted and cells were rinsed with PBS followed by the addition of 500 μL 10 μm ROS probe solution, diluted by the medium without FBS, and another 2 h incubation at 37°C. Stained cells were digested by trypsin and resuspended in 200 μL PBS for flow cytometry to detect cell apoptosis. Meanwhile, the inhibition rate (%) = (F_control group_ − F_sample group_)/F_control group_ × 100%

Where the F_sample group_ is the fluorescence intensity of the sample group (Fer‐1 group or Ptdser‐NM‐Lipo/Fer‐1 group); F_control_ is the fluorescence intensity of the control group.

### 2.6 Cytotoxicity test

RAW264.7 cells in the logarithmic growth phase were seeded in a 96‐well plate at a density of 8000‐1000 per well for 24 h and divided into four groups: Blank group (PBS), Control group (DMEM), Fer‐1 group, and Ptdser‐NM‐Lipo/Fer‐1 group (at the Fer‐1 concentration of 0, 1, 2, 3, 5, 7, 11 μmol/L). After 24 h or 48 h incubation, the medium was aspirated and cells were incubated with 200 μL 0.5 mg/ml 3‐ (4,5)‐dimethylthiahiazo (‐z‐y1)‐3,5‐di‐ phenytetrazoliumromide solutions for 4 h at 37°C. Cells were added 200 μL methyl sulfoxide to dissolve the formazan crystals and the absorbance of plates was measured at 570 nm by a microplate reader to determine the cell viability. Cell viabilities were calculated according to the formula given below:

$$Cell\;viability\;\left(\%\right)=\left(A_{sample}-A_{blank}\right)/\left(A_{control}-A_{blank}\right)\times 100\%$$

Where A_sample_ is the absorbance of the sample group; A_control_ is the absorbance of the control group; A_blank_ is the absorbance of autozero.

### 2.7 Assessment of targeting

This study was approved by the Ethics Committee of China Pharmaceutical University and the animal experiments were performed according to the Guide for the Care and Use of laboratory animals. ApoE^−/−^ mice (6 weeks old, 18–22 g) were seeded with a high‐fat and high‐cholesterol diet for 15 weeks to conduct AS model. The mice were divided into four groups (*n* = 3): Free DiR Group、DiR/NM‐Lipo Group、DiR/platelet membrane (PM)‐Lipo Group、DiR/PtdSer@NM‐Lipo Group (0.3 mg/kg DiR). The DiR was encapsulated in liposomes through the thin‐film dispersion method. The formulations were injected into the AS model mice and the mice were sacrificed after 6 h. The aorta and other organs were harvested and imaged by in vivo imaging system (IVIS, PerkinElmer) to detect the fluorescence intensity.

## 3 RESULT

### 3.1 Synthesis and characterisation of PtdSer‐NM‐Lipo/Fer‐1

**FIGURE 1:**
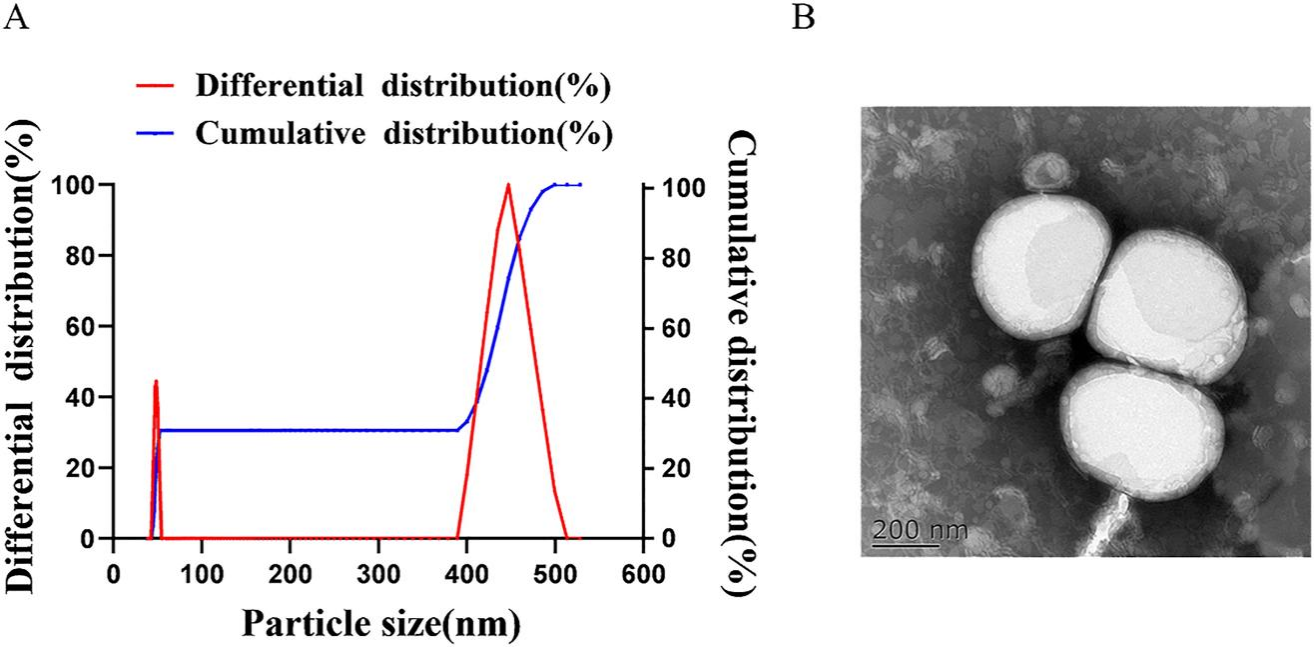
(a) The particle size of PtdSer‐NM‐Lipo/Fer‐1 (b) the morphology of PtdSer‐NM‐Lipo/Fer‐1 observed by transmission electron microscopy (TEM) (scale bar = 200 nm).

In the synthesis, PtdSer‐NM‐Lipo/Fer‐1 was formed by extruding NM and liposomes together. Based on the NM taking on the role of lipid building blocks that merge with phosphatidylcholines to generate stable liposomes rather than a coating shield to cover the surface, the hydrodynamic diameter was 174.9 ± 16.89 nm (Figure 1a), which was similar with the diameters shown on the TEM and the morphology of PtdSer‐NM‐Lipo/Fer‐1 was spherical (Figure 1b). Meanwhile, the zeta potential of PtdSer‐NM‐Lipo/Fer‐1 was −15.6 ± 0.346 mv, which indicated the successful fusion of membrane and liposome.

#### 3.1.1 ROS‐cleaning effect on MOVAS cell

The fluorescence intensity was used to gauge the ROS level in H_2_O_2_‐treated MOVAS cells. When H_2_O_2_‐solution was added, cells in the H_2_O_2_ group became visibly wrinkled and deformed, showing that the rate of cell necrosis or apoptosis increased dose‐dependently with H_2_O_2_ concentration. When the concentration of H_2_O_2_‐was 250 μmol/L, the background ROS level in the H_2_O_2_ group was quite low, pointing to the meagre stimulation of 250 μmol/L H_2_O_2_. Notably, the irritation of 500 μmol/L H_2_O_2_ caused the green fluorescence to activate, showing a progressive buildup of ROS. When the H_2_O_2_ concentration reached to 1000 μmol/L, the cells float in the medium and have a low survival rate. Therefore, 250 and 500 μmol/L of H_2_O_2_ were chosen.

**FIGURE 2:**
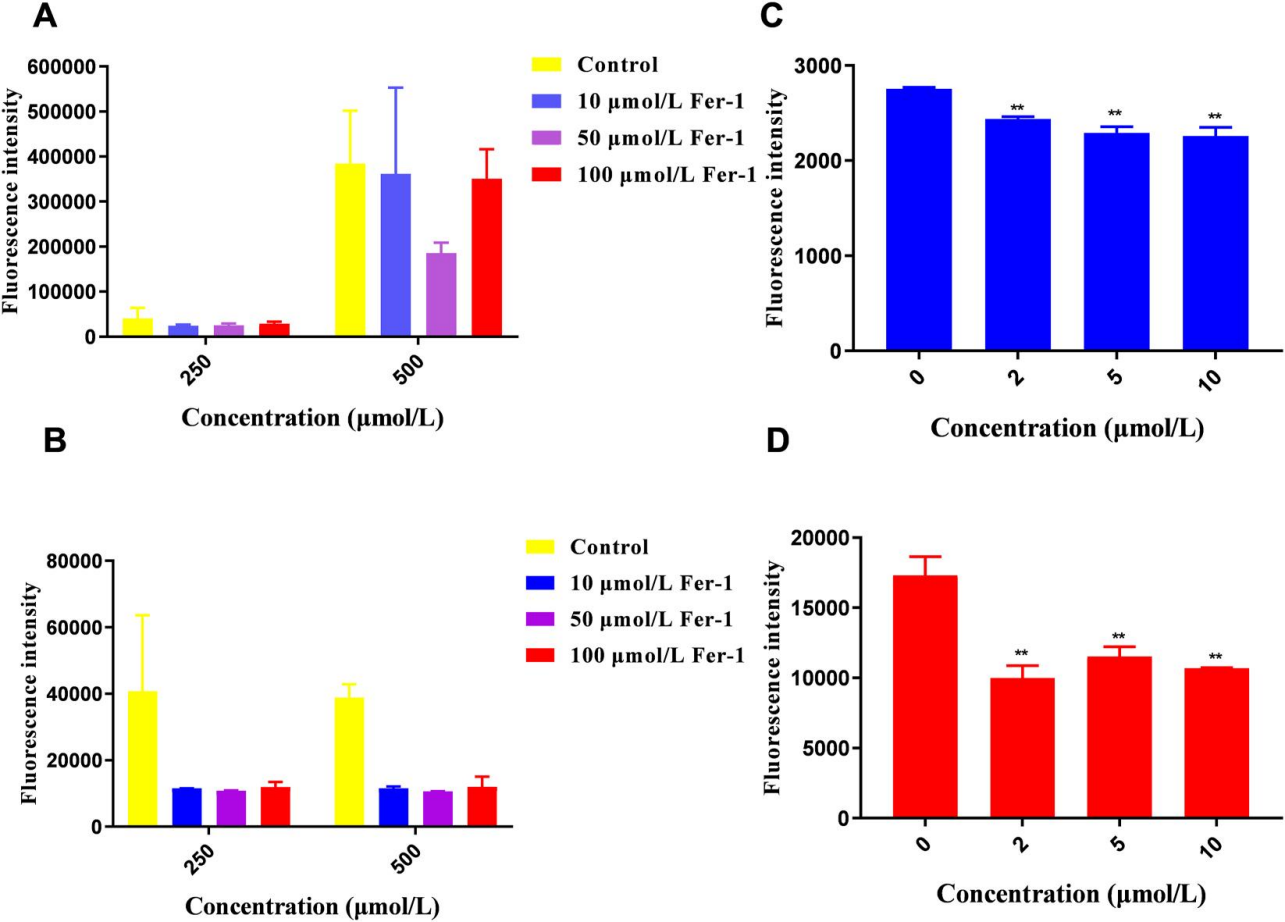
ROS‐cleaning effect of Fer‐1 in H_2_O_2_‐MOVAS cells in different groups: (a) the cell fluorescence intensity of Control group (DMEM medium) and Fer‐1@ H_2_O_2_ group (with the Fer‐1 concentration of 10,50 and 100 μmol/L), cells were stimulated with different concentration of H_2_O_2_ for 24 h followed by the addition of Fer‐1 solution for another 24 h; (b) the cell fluorescence intensity of control group and Fer‐1& H_2_O_2_ group (with the Fer‐1 concentration of 10,50 and 100 μmol/L); For compare, the ROS‐cleaning effect of Fer‐1 group and Ptdser‐NM‐Lipo/Fer‐1 group in LPS‐induced RAW264.7 cells: (c) The cell fluorescence intensity of control group and Fer‐1 group (with the Fer‐1 concentration of 2, 5, 10 μmol/L) (d) The cell fluorescence intensity of control group and Ptdser‐NM‐Lipo/Fer‐1 group (with the Fer‐1 concentration of 2, 5, 10 μmol/L). LPS, lipopolysaccharide.

Figure 2a indicated that different concentrations of Fer‐1 solution had no discernible impact on lowering the ROS level in the cells induced by 250 μmol/L H_2_O_2_, which was speculated to be the reason for the low amount of ROS production in cells. To test the validity of this hypothesis, Fer‐1 solution was added to the cells induced by 500 μmol/L H_2_O_2,_ the green fluorescence intensity was reduced significantly compared with a control group, and the level of ROS showing the greatest suppression at a Fer‐1 concentration of 50 μmol/ml, suggesting the role of Fer‐1 in reducing oxidative stress and scavenging ROS when sufficient ROS are produced.

Based on the time‐dependence of H_2_O_2_‐induced oxidative stress, Fer‐1& H_2_O_2_ group was established to prevent the impact of cell apoptosis or necrosis after 24 h simulation on the production of ROS in cells and the reversal effect of the Fer‐1 solution. Figure 2b showed that the fluorescence intensity in the control group was 4‐fold higher than that in the Fer‐1 treated cells when cells were induced by 250 μmol/L H_2_O_2_, and the ROS‐cleaning efficacy of Fer‐1 in various concentrations was comparable and reached a maximum at 50 μmol/L, suggesting that Fer‐1 inhibited the H_2_O_2_‐mediated generation of ROS and the highest antioxidant efficacy.

#### 3.1.2 ROS‐cleaning effect on RAW264.7 cells

LPS is a major constituent of gram‐negative bacteria and has been reported to be a classic stimulator in atherosclerosis‐induced inflammation. After LPS‐induced RAW264.7 cells were incubated with Fer‐1 (Figure 2c), the level of ROS was inhibited by the addition of Fer‐1 solution in a dose‐dependent manner, suggesting that Fer‐1 may efficiently consume intracellular ROS, inhibit ferroptosis, and guard against lipid peroxidation to lessen the degree of GPX4‐dependent lipid ROS buildup in total ROS level. Meanwhile, in contrast to the ROS‐cleaning effect in the Fer‐1 group (Figure 2d), the ROS inhibition rate of Ptdser‐NM‐Lipo/Fer‐1 was 38.26% which was significantly higher than that of free Fer‐1 whose inhibition rate was 18.00% at the Fer‐1 concentration of 10 μmol/L, which might attribute to that Ptdser‐NM‐Lipo/Fer‐1 was taken up by the induced macrophages based on the tropism and affinity of neutrophil membranes to the inflammation.

### 3.2 Cytotoxicity test

**FIGURE 3:**
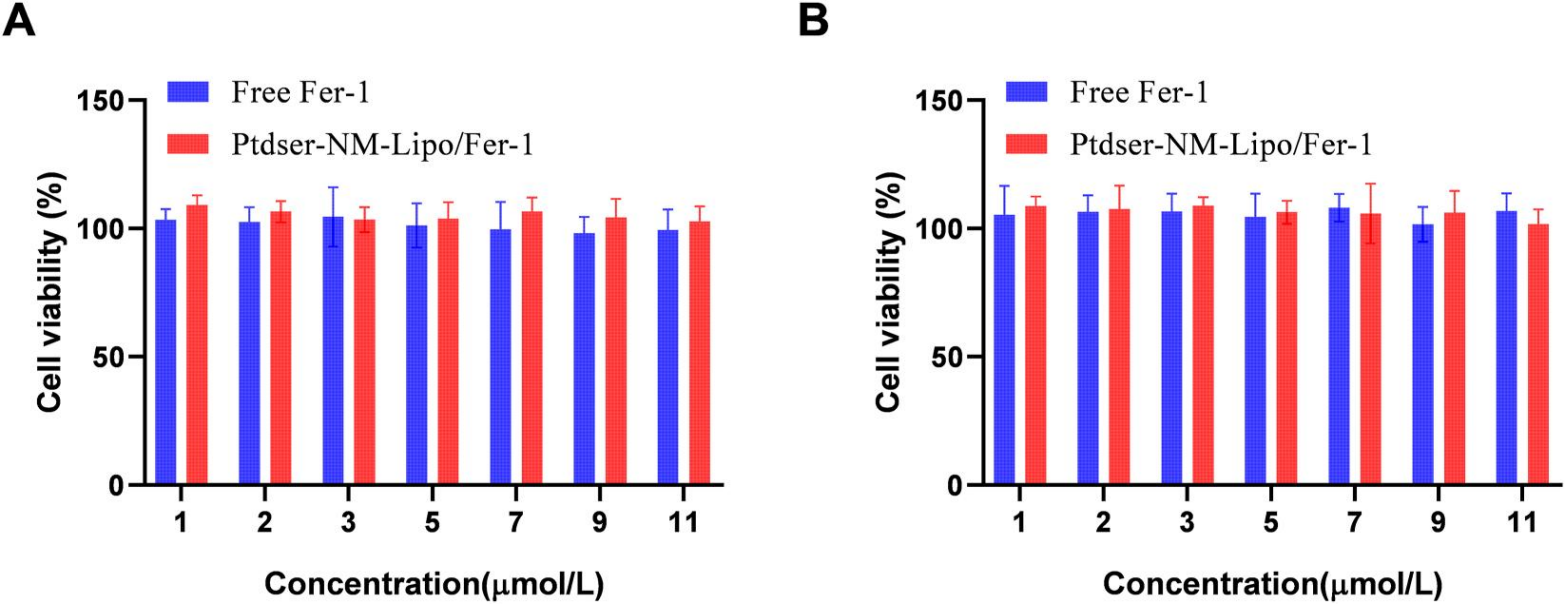
Cell viability of RAW 264.7 cells after co‐incubating with Fer‐l solution and Ptdser‐NM‐Lipo/Fer‐1 for 24 h (a) and 48 h (b).

Figure 3 showed that the RAW264.7 cell viability was more than 90% after the pre‐treatment with Fer‐1 solution and Ptdser‐NM‐Lipo/Fer‐1 in the concentration range of 1–11 μ mol/L for 24 and 48 h, indicating that the formulation was safe for RAW246.7 and the low toxicity or low‐risk from Ptdser‐NM‐Lipo/Fer‐1 for the treatment of atherosclerosis.

### 3.3 Assessment of targeting

To evaluate the targeting ability and biodistribution, NM‐Lipo, PM‐Lipo, and PtdSer@NM‐Lipo were encapsulated with DiR and injected intravenously into the induced ApoE^−/−^ mice. The targeting ability was evaluated by fluorescence intensity of the arteries and in vivo distribution was studied by fluorescence intensity of organs.

**FIGURE 4:**
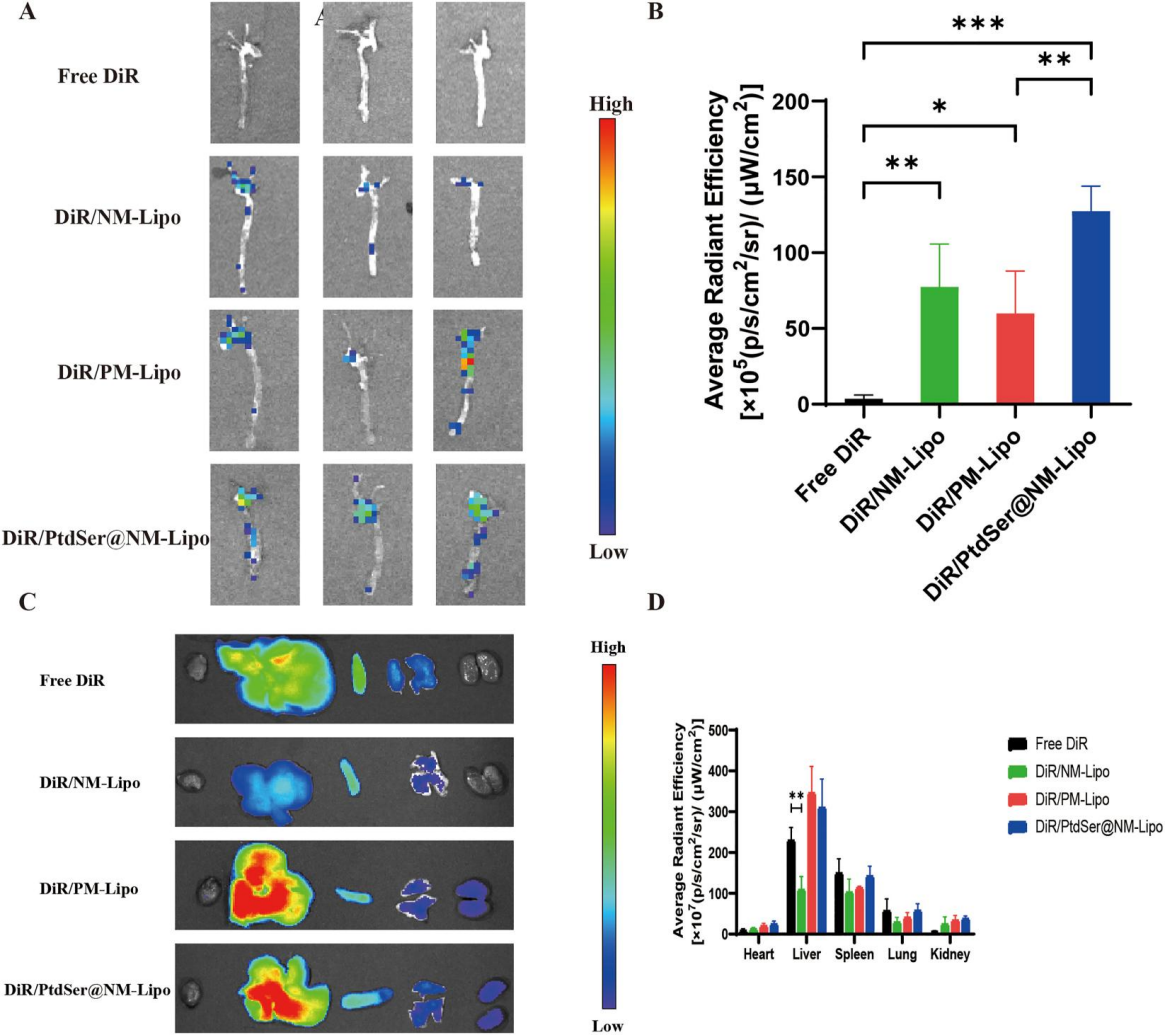
(a) The representative images of ex vivo fluorescence images and (b) quantitative data illustrating the accumulation of DiR fluorescent signals in the aorta. Mean ± SD, *n* = 3. (c) The representative images of ex vivo organs of mice at 6 h after injection of different groups. (d) The average fluorescence intensity of ex vivo organs of mice at 6 h after injection of different groups. Mean ± SD, *n* = 3.

In contrast to the control group (Figure 4a and b), NM‐Lipo displayed a 1.3‐fold greater radiant efficiency than that of PM‐Lipo and PtdSer@NM‐Lipo displayed a 1.6‐fold greater radiant efficiency than that of NM‐Lipo, respectively, indicating that NM‐Lipo, PM‐Lipo, and PtdSer@NM‐Lipo displayed selective aggregation at atherosclerotic plaques and a higher fluorescence signal, implying greater targeting ability and enhanced adherence to aortic plaque.

For the NM‐Lipo group, the smaller size of NM‐Lipo improved the membrane diffusion into the plaque and the aggregation at plaques demonstrated that the NM preserved the chemotaxis on the site of inflammation. For the PM‐Lipo group, the membrane‐bound complement regulator proteins (collagen and fibrin) on the surface of damaged endothelial cells promoted the recruitment of platelet membranes to plaque sites. Meanwhile, the NM‐Lipo group exhibited significantly enhanced accumulation and fluorescence intensity compared with the PM‐Lipo group, indicating that the targeting on plaques was membrane‐type‐specific and the NM‐Lipo group process better‐targeting ability.

On the other hand, when NM was modified with PtdSer, it became capable of being absorbed by liver phagocytes, and had a stronger chemotactic effect on plaque, indicating the excellent multivalent targeting properties of Ptdser‐NM‐Lipo. Notably, the aorta in PtdSer@NM‐Lipo injected mice displayed the highest fluorescence intensity when compared to the other three groups, indicating that more PtdSer@NM‐Lipo had accumulated in the aorta. Based on PtdSer can be recruited to inflammatory sites and recognised by phagocytes, it was demonstrated that PtdSer@NM‐Lipo has a tendency to aggregate at the plaque and the enhanced multivalent targeting properties following modification of Ptdser and the NM.

As in Figure 4c and d, based on the same fluorescence loading, lower accumulation of NM‐Lipo was noted in the liver and spleen compared with the DiR group, which might be due to the presence of self‐tolerance proteins, good immune evasion, and the ability of the polyethylene glycol chain to significantly lessen hepatic reticuloendothelial system phagocytosis. In contrast, the fluorescence intensity in the PM‐Lipo group was significantly higher in the liver and spleen than in other groups, indicating that PM‐Lipo was more accumulated in the liver and spleen which might be because the vWF factor receptor complexes on the platelet surface could be recognised and phagocytosed by liver macrophages [17]. All of the results indicated that the modification of NM or PM and PtdSer could enhance the aorta vascular plaque‐targeting which might present a promising strategy for AS treatment.

## 4 DISCUSSION

Atherosclerosis is a chronic inflammatory disease in which endothelial dysfunction is a key initial event. In atherogenesis, ROS can cause lipid peroxidation on the surface of macrophages and significantly increase the accumulation efficiency of cholesterol on the surface of macrophages, resulting in a more rapid conversion of macrophages into foam cells and an accelerated process of atherogenesis. Therefore, scavenging intracellular ROS is the direction of the research.

In the cellular ROS scavenging assay, free Fer‐1 solution and Ptdser‐NM‐Lipo/Fer‐1 were taken up by macrophages through passive diffusion and endocytosis, respectively. However, the Ptdser‐NM‐Lipo/Fer‐1 group displayed a greater ROS inhibition rate than free Fer‐1 at the same concentration, which might be due to the fact that cells under oxidative stress are prone to take up more Ptdser‐NM‐Lipo/Fer‐1 based on the membrane fusion. In response to similar inhibition rates at different concentrations of Ptdser‐NM‐Lipo/Fer‐1, which might be due to possible saturation of the binding site of the nuclear factor proteins at the concentration of Fer‐1 used in the experiment. Therefore, to increase the therapeutic effect and lessen systemic toxic side effects, the NM biomimetic delivery system (Ptdser‐NM‐Lipo/Fer‐1) can precisely deliver Fer‐1 to macrophages at the site of the atherosclerotic plaque, improve its solubility and drug load in vivo, prolong sustained release, and reduce its non‐specific distribution.

In the present study, Ptdser‐NM‐Lipo/Fer‐1 preferentially accumulated in the atherosclerotic plaque, demonstrating an effective targeting ability of Ptdser‐NM‐Lipo/Fer‐1. Previous studies have shown that neutrophils are attracted to inflammatory sites and aggregate at atherosclerotic plaques, making it possible to effectively target atherosclerotic plaques by simulating neutrophil recruitment. In contrast to earlier studies, where ligands were used to modify the surface of particles to bind to the overexpressed antigen at the disease site, NM nanoparticles offered an opportunity to successfully deliver anti‐inflammatory drugs to the atherosclerotic site by capitalising on the inflammatory tendency of neutrophils and the covalent targeting effect with Ptdser to achieve high positioning and precise drug delivery to the inflammatory site. Meanwhile, it has the potential to postpone the process of atherosclerotic plaque rupture and improve the prognosis of patients with atherosclerosis by improving the inflammatory microenvironment of plaque. Therefore, Ptdser‐NM‐Lipo/Fer‐1 is a promising carrier for atherosclerosis treatment.

## 5 CONCLUSION

In the study, we prepared a NM‐based biomimetic delivery system (Ptdser‐NM‐Lipo/Fer‐1) for precisely targeted therapy of AS by combining natural cell membranes with Ptdser‐modified liposomes. Ptdser‐NM‐Lipo/Fer‐1 displayed superior physicochemical characteristics, safety, and excellent ROS‐cleaning effect. In vivo, Ptdser‐NM‐Lipo/Fer‐1 served as a targeted delivery platform that preferentially accumulated in atherosclerotic plaques. Therefore, Ptdser‐NM‐Lipo/Fer‐1 is a promising targeting agent for atherosclerosis.

## AUTHOR CONTRIBUTIONS

Wei Li designed, performed the experiments, and wrote the manuscript. Chang Liu performed the experiments and edited the manuscript. Sichuan Wang and Naifeng Liu participated in designing experiments and editing articles. All authors have read and agreed to the published version of the manuscript.

## CONFLICT OF INTEREST STATEMENT

All the authors declare no potential conflict of interest relevant to this article.

## PERMISSION TO REPRODUCE MATERIALS FROM OTHER SOURCES

None.

## Data Availability

The datasets used and/or analysed during the current study are available from the corresponding author upon reasonable request.
